# The Evolution of a Civic Medical Service

**Published:** 1908-09-26

**Authors:** 


### Public Health and Hygiene.

### THE EVOLUTION OF A CIVIC MEDICAL SERVICE.

By A MEDICAL OFFICER OF HEALTH.

There are people so blind to the significant changes of their time, even in the matters which most nearly concern and interest them, that they fail utterly to perceive a trend of social movement that is surely and irresistibly sweeping away the little idols of opinion to which they cling as to the very foundations of social structure. Scarcely more than fifty years ago there were appointed to some of our larger cities the first medical officers of health known to this country. Just over thirty years ago the appointment of these officers became a statutory duty of every local sanitary authority. From the passing of the Public Health Act of 1875 down to that of the Education (Administrative Provisions) Act of last year no measure strikingly affecting the nature and expansion of the duties of the office served to call attention to the tremendous potentialities of which these appointments would seem to be the destined germ.

No greater tribute to the efficiency of the work of the medical officer of health could be paid than that which is ceded in the general acceptance of his office as the nucleus out of and around which a new order in the organisation of medicine is developing. His work has gone on silently and unobtrusively; to those with limited vision it has amounted to little more than decoration of the work of the sanitary inspector, itself supposed to consist in a somewhat exclusive preoccupation in the subject of "drains." To the student of sociology, however, the medical officer of health has revealed that hygiene is an urgent rudimentary social need to be attained by social changes quite as much as by sanitary reform in the mere externals of man's living places. And so we find that from a compiler of a mere statistical statement of births the medical officer of health is now actively concerned in the health progress of the children born. Whatever is capable of amelioration by public control affecting the ante-natal conditions of the child, it is his business to investigate; the training of the mother in infant nurture, the protection of infants from the adverse influences which our complex life renders so difficult of restraint, the purity and cleanliness of milk supply, the sufficiency of fresh air in the home, even the insistence on domestic cleanliness, and still more intimate domestic habits are duties which have been assumed from the inexorable pressure to which the social circumstances of the time have subjected him.

To the same forces he owes it that the new dispositions for the care of the health of the school child, necessitated by the Education (Administrative Provisions) Acts have centred in his office. Of so far reaching a character are these, that it is already seen that they go far to establish a public medical service which needs only to grow in order to replace the old *régime* of private practice. Judging from the open secrets as to the leanings of the Poor-law Commission, the probabilities are not that these anticipations will be falsified, but that in a few years the most profound changes in the order of medical practice will be established. The bearing of medicine upon life is continuous. From the cradle to the grave is incorrect description, for there is no moment in the life history of a human being at which medical interest may be said to commence, and death itself serves but to reveal how this same interest is more than personal, for it survives the fact of death and is essentially social.

The public have not been quick to learn that what is called State medicine is not a branch but a whole which includes many branches. The natural history of disease carries the physician at every turn to the social habits and circumstances upon which to so large extent disease is conditioned. Medicine has been so much more effectively engaged when she has been a State servant that the wonder is that education should have received a prior recognition at the hands of the State, while medicine was left to achieve its triumphs supported only by the generous grants of private charity and the somewhat sordid spoils of private practice.

What had the efforts of the practitioner availed in the magnificent achievement of practically abolishing such dread scourges as small-pox and typhus, but for the happy, if somewhat inadequate association of medicine with the State? The solid economic assets which have been given to the State as a direct result of medical knowledge and practice are inestimably great, and were but a small proportion of this gain set aside for the remuneration of the medical profession the efficiency of its work would be vastly increased.

Whether medicine is to be socialised, as it is called, or not depends upon forces mainly outside the profession, upon public opinion and the needs of the community. But both inside and outside the profession the question is formulating itself and the answer is shaping in more definite form than is yet fully appreciated.

In *New Worlds for Old*, Mr. H. G. Wells, ever sympathetic with the medical profession, writes strenuously in favour of the changes he foresees. "If there is one profession more than another in which devotion is implied and assumed, it is that of the doctor. . . . A practising doctor should be in lifelong perpetual war against pain and disease, just as a campaigning soldier is continually alert and serving. But existing conditions will not permit that. Existing conditions require the doctor to get his fee at any cost; if he goes about doing work for nothing they punish him with shabbiness and incapacitating need, they forbid his marriage or doom his wife and children to poverty and unhappiness. A doctor *must* make money, whatever else he does or does not do; he must secure his fees. He is a private adventurer, competing in a crowded market for gain, and keeping his energies perforce for those who can pay best for them. To expect him to behave like a public servant whose income and outlook are secure, or like a priest whose church will never let him want or starve, is ridiculous. If you put him on a footing with the greengrocer and coal merchant, you must expect him to behave like a tradesman. . . . It should be a part of the organisation of a civilised State to have a public health service of well-paid, highly-educated men distributed over the country, and closely correlated with public research departments, and a reserve of specialists who would be as ready and eager to face dangers and to sacrifice themselves for honour and social necessity as soldiers and sailors. I believe every honourable man in the medical profession under forty now would rather it were so. It is, indeed, a transition from private enterprise to public organisation that is already beginning.

"We have the first intimation of the change in the appearance of the medical officer of health, underpaid, overworked, and powerless though he is at the present time. It cannot be long before the manifest absurdity of our present conditions begins a process of socialisation of the medical profession entirely analogous to that which has changed three-fourths of the teachers in Great Britain from private adventurers to public servants in the last forty years."

That the existing conditions of practice are unsatisfactory no one knows better than the general practitioner. Few there are who have not occasion to regret the fact that their calling dooms them to almost intolerable conditions of working. Medicine is full of interest, its practice is humanising and absorbing, but as a means of livelihood it is well nigh unbearable. This I believe to be the silent verdict of the profession.

The public have cared little for the well-known hardships of the practitioner and done less. These have appealed to the community as *his* hardships to be grinned at and borne with along with the other inevitables of life. And it is less concern for the lot of the practitioner than need for his services that moves to his socialisation. There is more work urgently called for in the common interest than all the medical men can accomplish. Much, very much, that is done, is done at the cost of the profession. Those who can pay for medical aid get it irrespective of the medical urgency of their claims, those who cannot, equally irrespective of their needs, largely go without. This is an anomaly to the outrageous character of which custom and apparent necessity have blunted susceptibility. Slowly it is being realised that for the mass of the people the services of medicine can be secured on terms analogous to those of the embryonic health service, which in proportion to its cost, has conferred such inestimable benefits. When the public, instead of the patient, pays the practitioner, the public will pay less and the practitioner will receive more, the practitioner will do less, and the public get more, simply because of the economy of organised effort, over the chaotic dissipation of inco-ordinated struggle.

The growth of the public health service is, indeed, one of the significant social phenomena of the time. Its unification with general medical practice is a growing necessity, less because of any divergence than because its expansion is an inevitable outcome of public need and opinion, and because with this expansion the old demarcations are merged and the provinces of the practitioner and the medical officer no longer defined. If it be urged that the distinction between these provinces has not yet disappeared, that treatment is still the prerogative of the private practitioner, it must be answered that even were this true, which it is not, the time is rapidly approaching when all semblance of such distinctive limitations must go. The disabilities of Poor-law medical relief, real or imaginary, cannot be sustained, the power of education authorities to make provision for the care of the health of school children is the expression of a principle which cannot stop at the child. Even did it do so the transference of the care of children's diseases from the practitioner to the medical officer would represent such a large absorption of the practitioner into the official ranks that the residual private practice would constitute a largely diminished and diminishing quantity in a semi-socialised medicine. I have touched on a large problem growing rapidly and urgently. I believe it will be greatly to the advantage of the medical practitioner that in the main he will be known to the future as the medical officer. A civil medical service, I think, will be the solution of many of his difficulties. To many this contingency may appear remote. I do not share this view, and I think that, whether in the near or the distant future, it is destined to come, and that it would be ostrich-like to ignore its presages.

